# Multiple Omics Analysis of the Role of RBM10 Gene Instability in Immune Regulation and Drug Sensitivity in Patients with Lung Adenocarcinoma (LUAD)

**DOI:** 10.3390/biomedicines11071861

**Published:** 2023-06-29

**Authors:** Liusheng Wu, Qi Liu, Xin Ruan, Xinyu Luan, Yanfeng Zhong, Jixian Liu, Jun Yan, Xiaoqiang Li

**Affiliations:** 1 Beijing Tsinghua Changgung Hospital, School of Clinical Medicine, Tsinghua University, Beijing 100084, China; wuliusheng852@126.com; 2 Department of Graduate School, Anhui Medical University, Hefei 230032, China; 3 Department of Thoracic Surgery, Peking University Shenzhen Hospital, Shenzhen 518036, China; liuqi990526@163.com (Q.L.); sherstar0001@gmail.com (X.R.); luanxinyu0916@126.com (X.L.); zhongyf9785@163.com (Y.Z.); liujx0417@163.com (J.L.)

**Keywords:** RBM10, lung adenocarcinoma (LUAD), RNA-binding protein, immunoregulation, drug sensitivity

## Abstract

Objective: The RNA-binding protein RBM10 can regulate apoptosis during the proliferation and migration of pancreatic cancer, endometrial cancer, and osteosarcoma cells; however, the molecular mechanism underlying lung adenocarcinoma is rarely reported. Recent studies have detected multiple truncated and missense mutations in RBM10 in lung adenocarcinoma, but the role of RBM10 in lung adenocarcinoma is unclear. This study mainly explored the immune regulation mechanism of RBM10 in the development of lung adenocarcinoma and its influence on sensitivity to targeted therapy drugs. Methods: The transcriptome data of CGAP were used to analyze the RNA-seq data of lung adenocarcinoma patients from different subgroups by using the CIBERSORT algorithm to infer the relative proportion of various immune infiltrating cells, and Spearman correlation analysis was performed to determine the gene expression and immune cell content. In addition, this study utilized drug trial data from the GDSC database. The IC50 estimates for each specific targeted therapy were obtained by using a regression method, and the regression and prediction accuracy were tested via ten cross-validations with the GDSC training set. An immunohistochemical test was performed on the samples of 20 patients with lung adenocarcinoma in the subcomponent analysis of immune cells, and the protein expression of RBM10 in lung adenocarcinoma tissues was verified by cellular immunofluorescence assays. Nucleic acids were extracted at low temperatures, and qRT-PCR was used to verify the expression levels of the mRNA of RBM10 in lung adenocarcinoma tissues and normal tissues (*p* < 0.05). Results: After screening and inclusion using a machine language, the results showed that RBM10 was significantly highly expressed in the lung adenocarcinoma tissues. The related signaling pathways were mainly concentrated in ncRNA processing, rRNA metabolic processes, ribosome biogenesis, and the regulation of translation. The qRT-PCR for 20 lung adenocarcinoma tissues showed that the expression of RBM10 in these tissues was significantly different from that in normal tissues (*p* = 0.0255). Immunohistochemistry analysis and cell immunofluorescence staining also confirmed that RBM10 was involved in the immune regulation of lung adenocarcinoma tissues, and the number of immune cell aggregations was significantly higher than that of the control group. RBM10 regulates B cell memory-CIBERSORT (*p* = 0.042) and B cell memory-CIBERSOTRT-abs (*p* = 0.027), cancer-associated fibroblast-EPIC (*p* = 0.001), cancer-associated fibroblast- MCPCounter (*p* = 0.0037), etc. The risk score was significantly associated with the sensitivity of patients to lapatinib (*p* = 0.049), nilotinib (*p* = 0.015), pazopanib (*p* = 0.001), and sorafenib (*p* = 0.048). Conclusions: RBM10 can inhibit the proliferation and invasion of lung adenocarcinoma cells through negative regulation and promote the apoptosis of lung adenocarcinoma cells through immunomodulatory mechanisms. The expression level of RBM10 affects the efficacy of targeted drug therapy and the survival prognosis of lung adenocarcinoma patients, which has a certain guiding significance for the clinical treatment of these patients.

## 1. Introduction

In cell mitosis, chromosomal separation errors lead to whole chromosome aneuploidy mutation in daughter cells, or DNA damage leads to chromosomal structural changes, resulting in phenomena such as gene translocation, deletion, inversion, and fracture, which are all referred to as genomic instability [1,2]. RBM10, one of the gene-unstable groups, is a significantly mutated gene in tumor cells. In addition, RBM10 also belongs to the RNA-binding motif protein family [3]. In the process of malignant tumor progression, the mutation frequency of RBM10 indirectly affects tumor proliferation and migration through different mechanistic pathways and plays an important role in the apoptosis process of malignant tumor cells [4].

A related study performed exon and transcriptome sequencing on 98 lung adenocarcinoma precursor lesions and 99 invasive adenocarcinoma tissue specimens and identified RBM10 as a significantly mutated gene in the progression of lung adenocarcinoma [5]. It is closely related to ARM-level copy number alterations and HLA loss of heterozygosity, among other phenomena, which also shows that RBM10 is involved in the regulation of the tumor immune environment. Another study [6] found that RBM10 can regulate the expression of many genes involved in DNA repair, cell metabolism, proliferation, migration, senescence, and apoptosis by regulating the transfer factor P53 [7,8,9]. Cancer and oncology genome mapping (CGA) showed that EGFR mutations were most common in female lung adenocarcinoma patients, while mutations in RNA-binding motif protein 10 (RBM10) were most common in male lung adenocarcinoma patients. RBM10 was first identified in 1995 in unclassified cDNA of human bone marrow, and it was located on the X chromosome p11.23 [10]. The RBM10 transcriptome is about 3.5 KB in length, divided into 24 exons, and can be translated into 930 amino acid proteins [11]. There are many potential mechanisms of RBM10 in tumor regulation, including immune regulation in the tumor microenvironment to activate p53 [12]. The anticarcinogenic properties of RBM10 are achieved in part by blocking MDM2-mediated ubiquitination and p53 degradation, and it has also been shown to inhibit tumor growth and proliferation by selectively altering selective splicing [13]. RBM10 is significantly overexpressed in many malignant tumors, and it inhibits the proliferation and clonal formation ability of A549 cells and enhances the sensitivity of A549 cells to the chemotherapy drug paclitaxel by inducing apoptosis. RBM10 is an RNA-binding protein that interacts with telomere regulatory proteins [14]. In one study, although RBM10 did not affect telomerase activity, it had significant inhibitory effects on telomere length [15]. In addition, we also found that RBM10 can upregulate the expression of E-cadherin, downregulate the expression of Vimentin, and inhibit the migration of renal cell carcinomas (RCC) [16].

Targeted drug therapy is currently one of the effective means used for the treatment of lung cancer, and it is widely used in the systemic treatment of advanced patients, preoperative neoadjuvant therapy, and postoperative adjuvant therapy, for example [17]. In the late stage of targeted therapy, tumor sensitivity to drugs is often reduced and drug resistance occurs, which is the main reason for chemotherapy failure. Unlike cross-drug resistance, direct and indirect drug resistance are the main causes of death in cancer patients [18]. Individualization of drug use in cancer patients is also a problem that is difficult to solve at present [19].

In this study, we obtained sequencing data from the TCGA database of lung adenocarcinoma patients with high expression of RBM10 in order to analyze patient sensitivity to targeted drugs. Through statistical analysis of the data, for the lung adenocarcinoma patients with positive RBM10 expression, the survival prognosis and drug sensitivity of the lung adenocarcinoma patients showed statistically significant differences. This also suggests that RBM10 is involved in the regulatory process for the mechanisms underlying resistance to targeted drugs for lung adenocarcinoma, which has important clinical significance for the effective survival of patients [20]. A number of studies have reported on RBM10 with respect to lung adenocarcinoma, colorectal cancer, pancreatic cancer, endometrial cancer, and the relationship between malignant tumors such as breast cancer, but most of the research has mainly focused on RBM10 in studies on the function and mechanism of action of tumors; thus, the effect of the tumor microenvironment on tumor immune regulation and drug sensitivity is unclear [21]. In addition to exploring the function and mechanism of RBM10 in the progression of lung adenocarcinoma, this study also focused on exploring the immune regulation mechanism of RBM10 and its influence on the sensitivity of targeted therapy drugs.

## 2. Materials and Methods

### 2.1. Data Download and Acquisition

We used information from the TCGA (https://portal.gdc.cancer.gov/, accessed on 1 January 2022), GEO (https://www.ncbi.nlm.nih.gov/geo/, accessed on 1 January 2022), ImmPort (https://www.immport.org/home, accessed on 1 January 2022), and GDSC (Genomics of Drug Sensitivity in Cancer) (https://www.cancerrxgene.org/, accessed on 1 January 2022) databases to download transcriptome data on lung adenocarcinoma and clinical data. These data include gene expression data, miRNA expression data, copy number variation data, and so forth. The downloaded data were transformed and organized. Perl (https://www.perl.org/, accessed on 1 January 2022) was used to run scripts (merge.pl and move.pl) to convert gene IDs into gene names in the data, and tumor tissue samples were grouped with normal tissue samples according to the instability of the genome. The corresponding mutation data were obtained.

### 2.2. Data Algorithmic Processing

In R software (V3.6.0), the “edgeR” package was used to identify the downloaded TCGA data of 361 samples of gene transcription and 107 clinical samples’ data, adjusted|logFC|>2 and corrected *p* < 0.05. In this study, the mutation frequency of unstable genomes was screened and sorted out, and then only 25% of the genes with the highest mutation frequency were included in the high mutation group (i.e., the unstable group of genomes), while 25% of the genes with the lowest mutation frequency were included in the low mutation group (i.e., the stable group of genomes). RBP genes were extracted from the downloaded data and analyzed at the same time to obtain the prognostic RBP-related genes of lung adenocarcinoma. In addition, we also downloaded the subcomponent data of immune cells from ImmPort and corrected the data.

### 2.3. Matrix Deconvolution Analysis of Targeted Drug Sensitivity

CIBERSORT is an R or web version tool for deconvolving the expression matrix of human immune cell subtypes based on the principle of linear support vector regression. For more chip expression matrices, to determine an unknown mixture containing similar cell types to the expression of the matrix, deconvolution analysis is superior to other methods (LLSR LLSR, PERT, RLR, MMAD, and DSA). This method is based on a known reference set and provides a gene expression signature set for 22 immune cell subtypes. We used CIBERSORT (https://cibersortx.stanford.edu/, accessed on 1 January 2022) to screen the samples by transforming the gene expression matrix into the matrix of immune cells, that is, the composition of the various subtypes of immune cells in the tumor tissue. For genomics data based on the largest drug database (GDSC—Genomics of Drug Sensitivity in Cancer) (https://www.cancerrxgene.org/, accessed on 1 January 2022), we used R software package “pRRophetic” to predict each tumor sample for targeted drug sensitivity. The IC50 estimate for each specific targeted therapy was obtained through regression analysis.

### 2.4. Grouping Analysis of Genomic Instability

The mutation data obtained and sorted were statistically divided into high-frequency mutation genomes (the gene instability group) and low-frequency mutation genomes (the gene stability group). In this study, TCGA data and GEO data were used for double validation. At the same time as constructing and verifying the prognostic model of the unstable genome, experimental group A, validation group B, and group C were used as the statistically comprehensive results of the two groups.

A Kaplan–Meier curve was used to evaluate the survival time of unstable genomes, and *p* < 0.05 indicated that the difference between the two groups was statistically significant. An AUC curve was frequently used to evaluate the feasibility and accuracy of the model, which was constructed with big data, and AUC > 0.6 indicated that the model had a good ability for prediction and evaluation.

### 2.5. Prognostic Model Construction and Survival Analysis of Prognostic RBP

The core module can be determined by calculating the correlation coefficient between the feature vector gene of each module and the sample feature information. Genomically unstable genes are a series of genes with high connectivity and modular connectivity. One of the goals of WGCNA is to find the key genes of the target modules. In general, the key genes in this submodule are biologically more important than in the global network. Module identity can be used to measure the importance of genes in a module, and it has been shown that M is positively related to module connectivity. Therefore, the major genes of a specific module are selected based on the M value. If the value of M in a specific module is the largest, the gene is considered to be a key gene.

### 2.6. GO and KEGG Pathway Enrichment Analysis

We used the application DAVID (https://david.ncifcrf.gov/summary.jsp, accessed on 1 January 2022), an online database, to carry out gene ontology for target mRNA genes in the ceRNA network. We also used GO annotation and Kyoto Encyclopedia of Genes and Genomes (KEGG) pathway enrichment analysis, with *p* < 0.05 as the screening condition. GO analysis included biological process (BP), cell component (CC), and molecular function (MF) analysis.

### 2.7. Construction of Protein–Protein Interaction (PPI) and Screening of Key Genes

Target mRNA genes in the chromosome instability group gene network were uploaded to the STRING online database (https://string-db.org/, accessed on 1 January 2022), a PPI network was constructed, medium confidence was taken as the truncation standard, and the nodes without mutual relationships in the network were hidden. The PPI network information obtained from the STRING database was imported into Cytoscape software (V3.8.2). The MCODE plug-in Cytoscape was used to screen the key modules and identify the seed factors at the same time, and the MCC algorithm in the CytoHubba plug-in was used to screen the hub genes.

### 2.8. PCR Assay Analysis

The expression of RBM10 in lung adenocarcinoma tissues and adjacent tissues was also verified by PCR assays. The real-time PCR results are generally presented in the form of the mean ± standard deviation. The results are usually analyzed by the method of relative halo analysis; that is, the 2^−ΔΔct^ method is used to obtain the expression level of the target gene, β-actin is the reference gene, and RBM10 is the target gene. The expression level of each gene is measured twice, so the average of the two groups of results is taken in the final Ct value. For the calculation of the ΔΔCt value, the final obtained samples and the normal control group 2^−ΔΔct^ were the differential expression multiples of the two groups.

### 2.9. Analysis of the Pathological Characteristics of Lung Adenocarcinoma

H&E staining is one of the common staining methods used in paraffin sectioning. Dehydration was carried out, and the transparency of the sections was determined. After H&E staining, the sections should be thoroughly dehydrated and transparent before they can be capped with neutral gum. If the dehydration process is not thorough, the film shows a white mist after sealing, is blurred under the microscope, and can easily fade. The sections were dehydrated with grade 1–2 absolute ethanol or xylene carbolic acid.

After staining with hematoxylin, the nucleus and calcium salt mucus were blue, and then eosin, a cytoplasmic dye, was used to stain the cytoplasm so that the different components of the cytoplasm appeared as different shades of pink. Through this, various tissue or cellular components and the general morphological and structural characteristics of the lesions can be shown.

### 2.10. Immunohistochemical and Cell Immunofluorescence Analysis

Paraffin sections (5 µm thick) extracted from the tissue were dewaxed in xylene and rehydrated in graded alcohol, and endogenous peroxidase activity was blocked by infiltration into 0.3% hydrogen peroxide. Next, the sections were rinsed with phosphate-buffered saline (PBS) (PH 7.2), and all of the sections were treated with immunoglobulin IgG as a secondary antibody. After being washed with PBS, the sections were incubated with DAB (0.02% diaminobenzidine tetrahydrochloric acid, 0.1% phosphate buffer, and 3% H) to observe the peroxidase reaction. The final sections were stained with hematoxylin, dehydrated with graded alcohol, and mounted on resin supports. The stained sections were examined with a microscope.

Isolated and cultured cells were fixed with 4% paraformaldehyde for 15 min. Next, the cells were blocked with 2% BSA in PBS and then treated with 0.2% triton for 15 min. The diluted primary antibodies were added as follows: anti-RBM10 (Abcam, 1:200, Boston, MA, USA) and then the secondary antibody (1:500, Invitrogen, Los Angeles, CA, USA). In addition, the cells were stained with DAPI (Sigma, St. Louis, MO, USA) and observed under a fluorescence microscope.

## 3. Results

### 3.1. Project Research and Analysis Process

**Figure 1:**
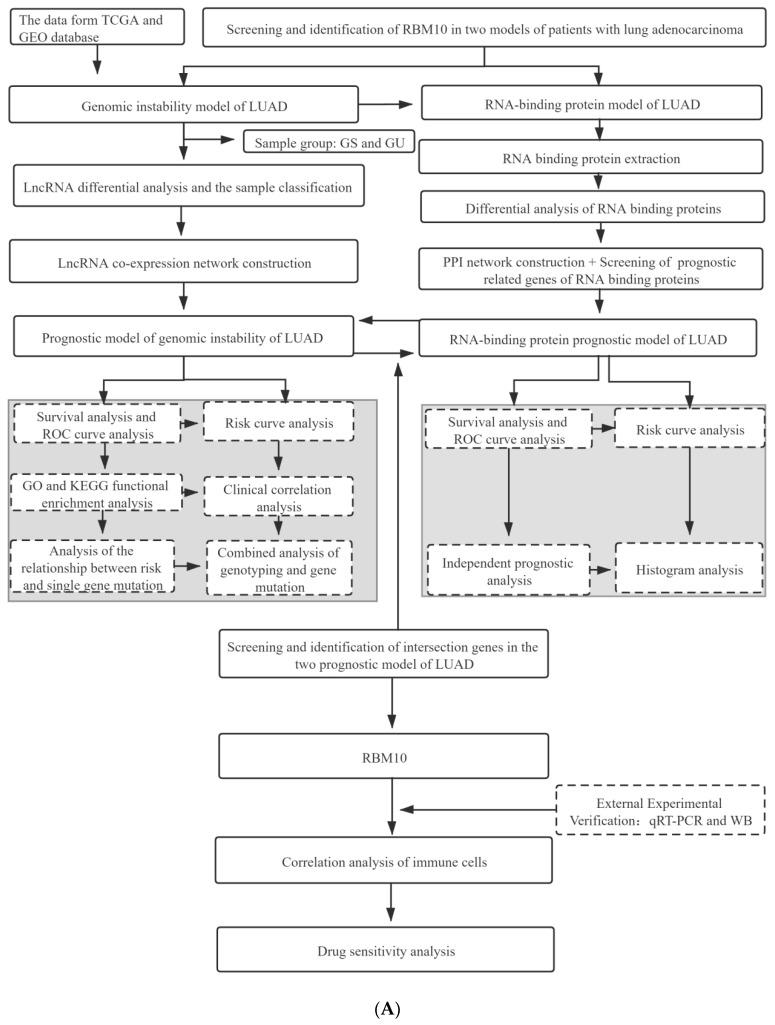

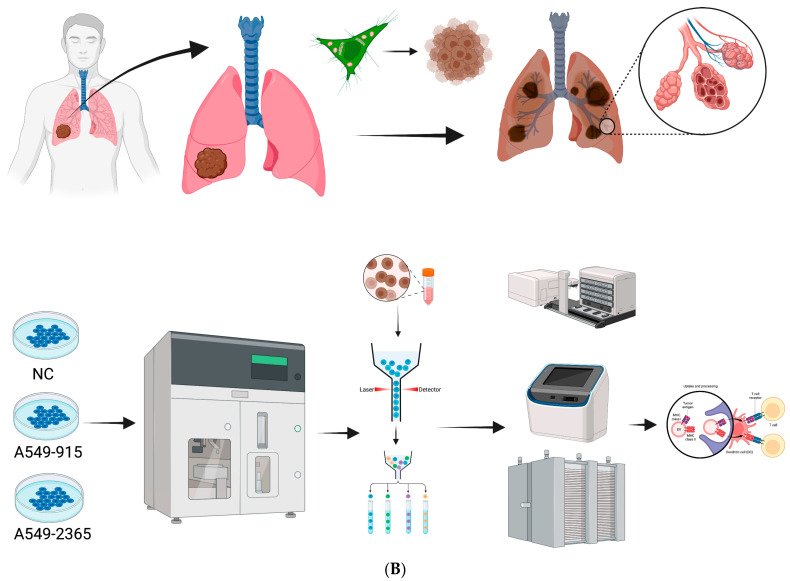
(**A**) Flow chart of the research project. (**B**) Flow chart of the in vitro experiment.

The data in this study were obtained from the TCGA and GEO databases. After screening and identifying the intersection of the lung adenocarcinoma gene set and the chromosome gene instability group gene set, a separate network was constructed to analyze the prognosis model of the lung adenocarcinoma chromosome instability gene set, and an ROC curve was used to evaluate the prediction accuracy of the prognosis model. In addition, univariate and multivariate analyses of gene clinical risk and functional enrichment analysis of related signaling pathways were further analyzed. In this study, another risk model of lung-adenocarcinoma-associated RNA-binding protein was constructed to analyze the distribution of upregulation and downregulation of differential genes and the interaction analysis of the PPI network. The second model was also evaluated by an ROC curve and a survival curve. In the later stage of this study, qRT-PCR assays, immunohistochemistry analysis, cell immunofluorescence assays, and pathological characteristics analysis were performed to investigate the mechanism of drug sensitivity and the immune microenvironment (Figure 1).

### 3.2. Identification of RNAs Associated with Genomic Instability in Patients with Lung Adenocarcinoma

**Figure 2:**
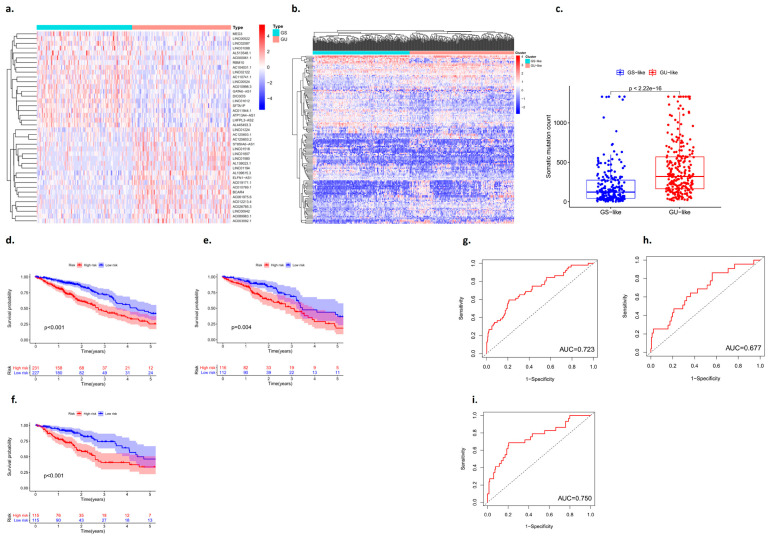
Identification of genes associated with genomic instability in lung adenocarcinoma. (**a**,**b**) Heat map analysis of the number of unstable gene mutations (GS: genomic stability; GU: genomic instability). (**c**) Somatic mutation count between the GS group and the GU group. (**d**–**f**) Kaplan–Meier curves analysis. (**g**–**i**) ROC curve analysis.

Based on the number of mutations carried by lung adenocarcinoma patients, the 25% of patients with the most and fewest mutations were defined as the GU group and the GS group, respectively, and the differentially expressed RNAs in the two types of samples were identified (Figure 2a,b). There was a statistically significant difference in the number of somatic mutations between the GU group and the GS group, and the GU-like group tended to carry more mutant genes (*p* < 0.001) (Figure 2c).

The data of lung adenocarcinoma patients from the TCGA were divided into a training group, a testing group, and the whole group. The prognostic genes in patients with lung adenocarcinoma were identified based on univariate Cox proportional hazards regression. The patients were divided into high- and low-risk groups, and the candidate genes were analyzed by multivariate Cox proportional hazards regression analysis. The Kaplan–Meier curves showed that there were statistically significant differences between the high-risk group and the low-risk group (*p* < 0.05) (Figure 2d–f). In addition, an ROC curve was used to analyze the prediction and accuracy of the three survival prognostic models constructed in this study. The ROC curve results showed that all three datasets had good predictive ability for the survival time of lung adenocarcinoma patients (AUC_1_ = 0.723; AUC_2_ = 0.677; AUC_3_ = 0.750) (Figure 2g–i).

### 3.3. Validation of the Prognostic Model with an Internal Dataset

**Figure 3:**
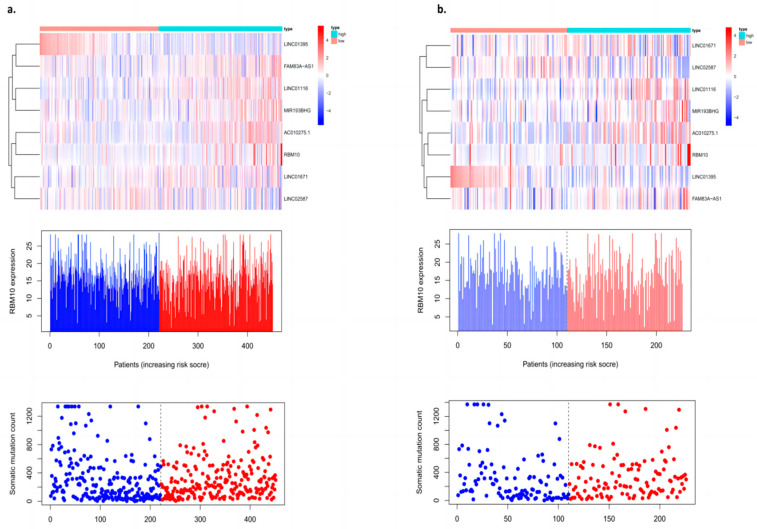
Prognostic model with an internal dataset ((**a**) training group and (**b**) test group).

The validation of the prognosis model of lung adenocarcinoma is mainly divided into two parts: One is the validation of the training group, and the other is the validation of the test group. The “Pheatmap” package in R software was used to organize the downloaded TCGA data and GEO data, calculate the risk coefficient of lung adenocarcinoma patients, calculate the distribution matrix of surviving and dead patients, analyze the differential expression of mutant genes in the prognostic heatmap related to immune infiltration, and successfully construct the prognosis model of lung adenocarcinoma. From left to right, we found that, with an increase in the mutation rate of mutated genes, the prognostic risk coefficient of lung adenocarcinoma patients also increased, and deaths were concentrated in the high-risk population area on the right, suggesting that the high mutation rate of prognosis-associated unstable genes may be associated with the high prognostic risk and mortality of lung cancer (Figure 3).

### 3.4. Analysis of RBM10 in Lung Adenocarcinoma Independent of Other Clinical Features

**Figure 4:**
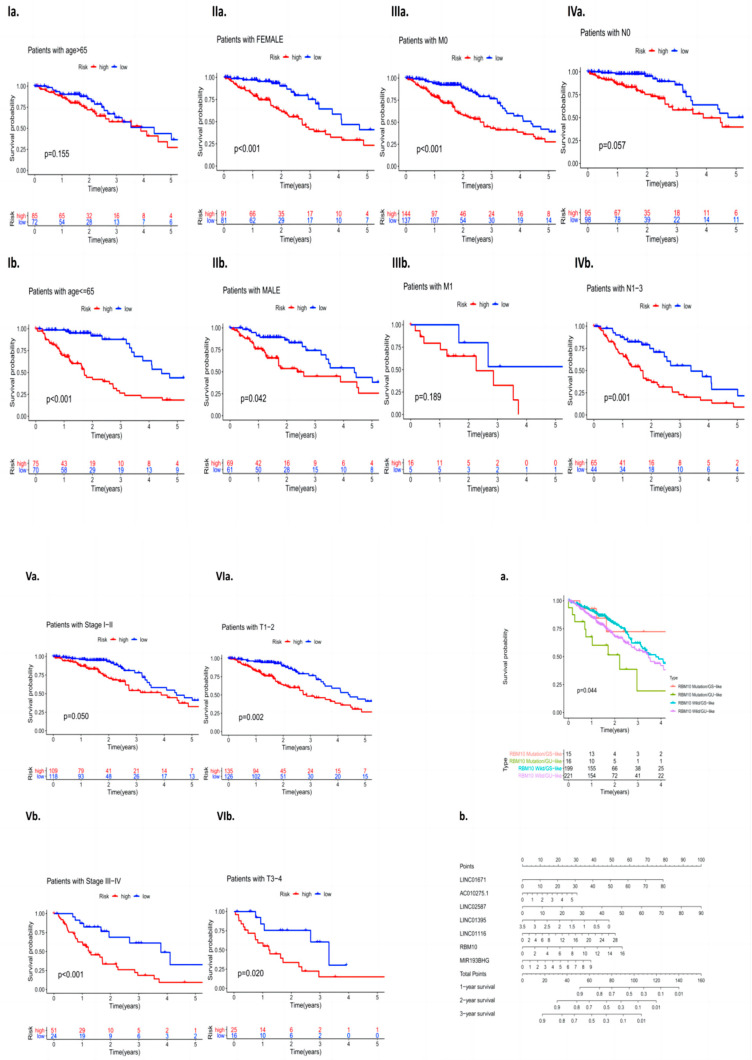
(**I**–**VI**): Independent analysis of clinical features. (**a**) Survival curve analysis of RBM10 and the wild-type mutations and (**b**) nomogram model analysis.

The clinical data of 572 patients included in the TCGA database (including gender, age, the clinical stage of the tumor, and whether there was distant metastasis) were divided into high- and low-risk groups according to the expression level of RBM10, and the independent predictive efficacy was evaluated. The survival curve results showed that there were statistically significant differences in the risk of some clinical factors in patients with RBM10 (+) lung adenocarcinoma, including age (*p* < 0.001), gender (female, *p* < 0.001; male, *p* = 0.042), M_0_ (*p* < 0.001), N_1–3_ (*p* = 0.001), stage I–II (*p* = 0.050), stage III–IV (*p* < 0.001), T_1–2_ (*p* = 0.002), and T_3–4_(*p* = 0.020) (Figure 4I–VI).

Survival curve analysis was performed on the clinical group data of lung adenocarcinoma patients with GS and GU classification of RBM10 and wild-type mutations. The results showed that there were still statistically significant differences among the four types of mutations (*p* = 0.044). A nomogram model of related RBM10 was constructed and showed that it can inhibit the occurrence and development of lung adenocarcinoma (Figure 4a,b).

### 3.5. PPI Network Analysis of Differential Genes

**Figure 5:**
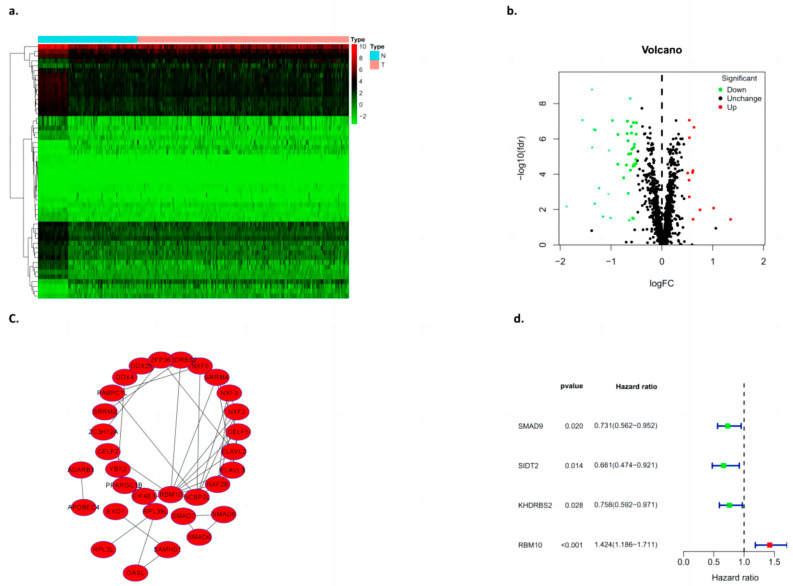
Construction of the differential gene PPI model. (**a**) Heat map clustering, (**b**) volcano plot, (**c**) PPI, and (**d**) single factor independent analysis of the forest map.

In this study, the expression of differential genes in lung adenocarcinoma tissues and adjacent tissues was analyzed by using a cluster heat map, and a volcano map was constructed according to the numbers of upregulated and downregulated genes (Figure 5a,b). Cytoscape software (V3.8.2) was used to construct the PPI network of deg, the Cytoscape plugin MCODE was used to obtain the most important module (Figure 5c), and several major hub genes (Including SMAD9, SIDT2, KHDRBS2, and RBM10) were further screened. At the same time, single factor independent analysis correction was applied (Figure 5d).

### 3.6. Functional Enrichment Analysis of the Algorithm Model

**Figure 6:**
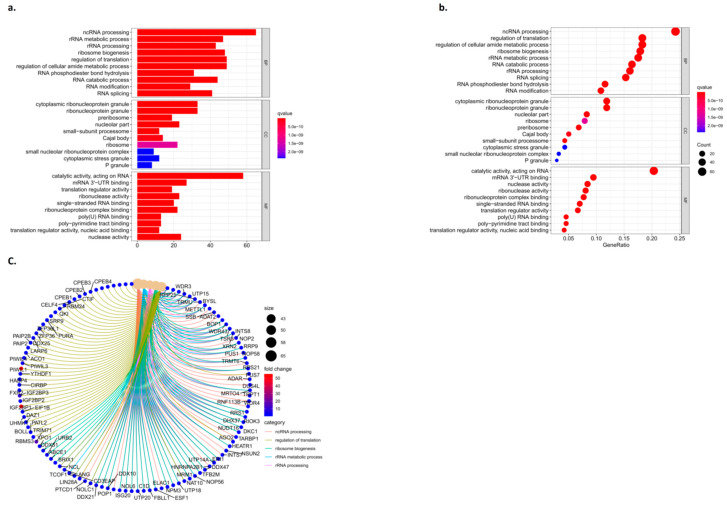
Results of the functional enrichment analysis. (**a**) Column graph of GO, (**b**) bubble pattern of GO, and (**c**) circle of the KEGG.

DAVID was used to further enrich the hub gene function and pathway. The GO analysis showed that the hub gene biological process (BP) item in ncRNA processing, rRNA metabolic processes, rRNA processing, the regulation of cellular amide metabolic processes, RNA catabolic processes, and RNA changes such as modification and RNA splicing increased significantly. CC: preribosome, nucleolar part, Cajal body, ribosome, P granule, etc. MF: catalytic activity, acting on RNA, mRNA3′-UTR binding, translation regulator activity, ribonuclease activity, single-stranded RNA binding, ribonucleoprotein complex binding, etc. (Figure 6).

### 3.7. Comparison of Survival Curves between the Test Group and the Control Group

**Figure 7:**
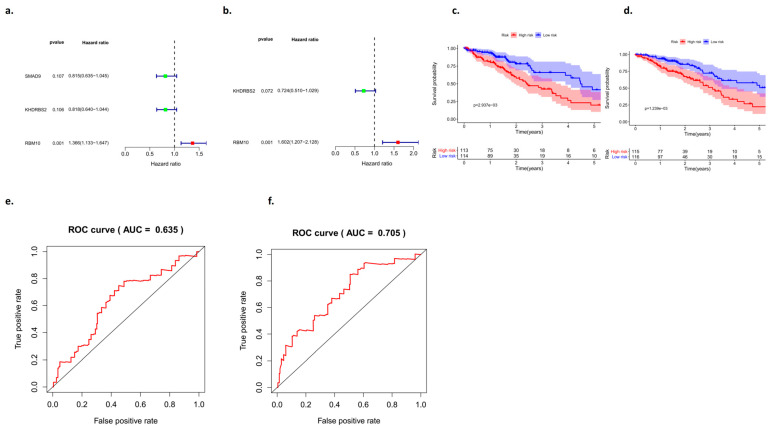
Analysis of the survival curves between the test group and the control group. (**a**,**b**) Univariate and multivariate analyses of the forest map, (**c**,**d**) survival curves, and (**e**,**f**) ROC curves.

In this study, the high expression of RBM10 was classified into a low-risk group, and the low expression of RBM10 was classified into a high-risk group. Univariate and multivariate independent analyses of survival and prognosis of lung adenocarcinoma were conducted using the data of the test group and the control group, respectively, and the results of both groups of data included RBM10 (Figure 7a,b).

Analysis of the survival curve results showed that there were significant differences between the test group and the control group in the high- and low-risk groups, which was of research value (*p* < 0.05) (Figure 7c,d), and an ROC curve was adopted to further verify our survival analysis results (AUC_test group_ = 0.635; AUC_control group_ = 0.705) (Figure 7e,f).

### 3.8. Risk Curve Analysis

**Figure 8:**
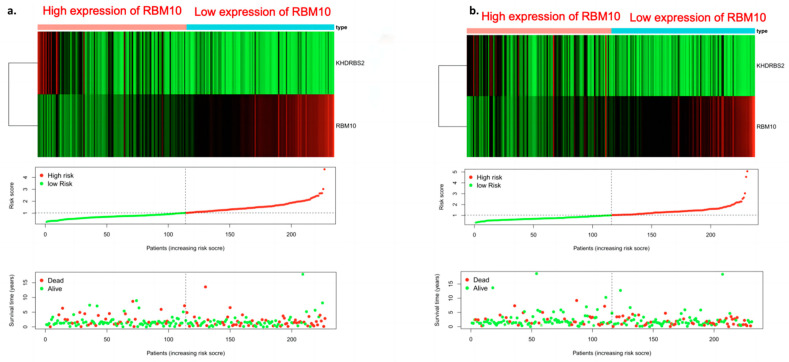
Survival prognostic risk curve, death case distribution matrix, and risk heat map of RBM10 expression in lung adenocarcinoma ((**a**) test group; (**b**) control group).

The “Pheatmap” package in R software was used to organize the downloaded TCGA data, calculate the risk coefficient of lung adenocarcinoma patients, calculate the distribution matrix of surviving and dead patients, analyze the differential expression of RBM10 in the immune-infiltration-related prognostic heatmap, and successfully construct the prognosis model of lung adenocarcinoma. From left to right, we found that, with the decrease in RBM10 expression, the prognostic risk coefficient of lung adenocarcinoma patients also increased, and deaths were concentrated in the high-risk population area on the right, which also proved that its prognostic correlation with low expression of RBM10 may be associated with the prognostic risk and high mortality of lung adenocarcinoma (Figure 8).

### 3.9. Analysis of Clinically Independent Prognostic Factors and Nomogram Analysis

**Figure 9:**
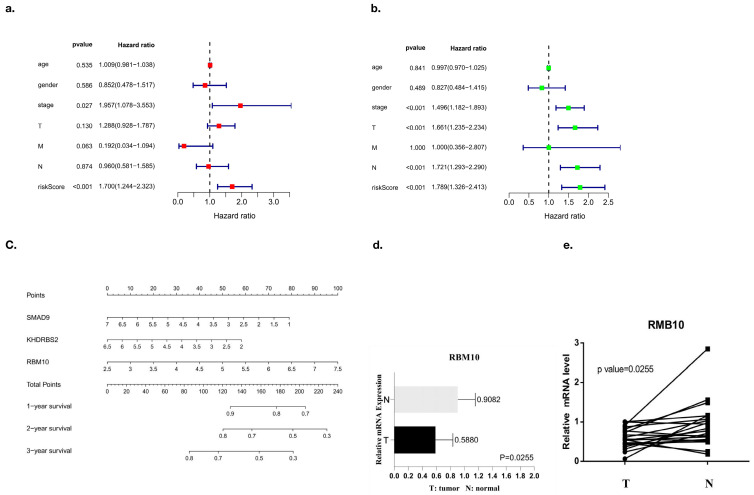
Prognostic analysis of independent clinical factors. (**a**,**b**) Univariate and multivariate analyses of clinical prognostic factors, (**c**) nomogram analysis, and (**d**,**e**) PCR results.

Factors related to the clinical prognosis of RBM10-positive patients were independently analyzed, and the forest map results showed that the prognostic factors of these patients were mainly related to their risk scores (*p* < 0.05) (Figure 9a,b).

Nomogram analysis was also used in this study, and the results also showed that SMAD9, KHDRBS2, and RBM10 had a certain risk correlation with the survival rate of lung adenocarcinoma patients (Figure 9c). Finally, the PCR results of RBM10 in cancer tissues and adjacent tissues of 20 clinical patients with lung adenocarcinoma were further compared for verification, supporting the conclusion that RBM10 has a negative regulatory effect on the survival and prognosis of lung adenocarcinoma (Figure 9d,e).

### 3.10. RBM10 Is Involved in the Immune Regulation of Lung Adenocarcinoma

**Figure 10:**
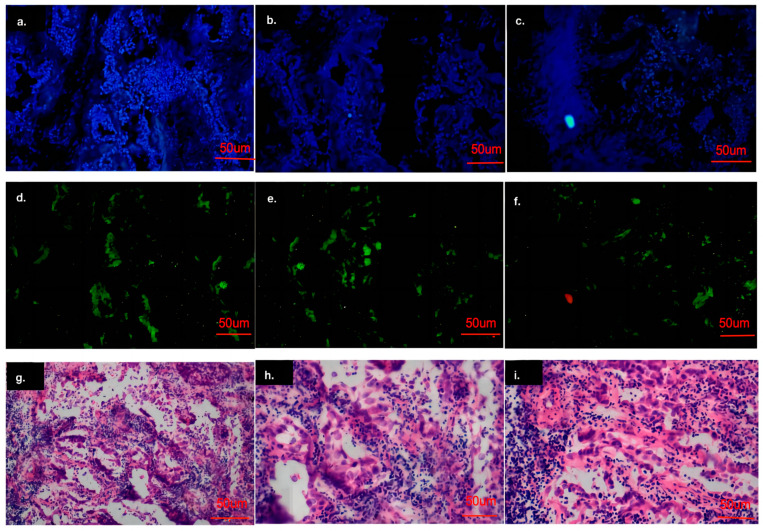
Analysis of the cell immunofluorescence experiment results. (**a**–**f**): Results of cell immunofluorescence experiment; (**g**–**i**): Results of immunohistochemical experiments.

The immunohistochemistry analysis showed that the cell nuclei of the carcinoma tissues were significantly enlarged and deeply stained, and RBM10 was expressed in the nucleus and cytoplasm (Figure 10a–f). The results suggest that the expression of the RBM10 protein in lung adenocarcinoma tissue decreased, which may be related to the negative regulation of the occurrence and development of lung adenocarcinoma.

Microscopically, adenocarcinoma is composed of new cubic and columnar cells, tending to form an adenoid structure supported by a fibrous matrix, with large or irregular nuclei, distinct nucleoli and mucin in the cytoplasm (Figure 10g–i).

### 3.11. Immune Cell Subcomponent Analysis and Drug Sensitivity Analysis

**Figure 11:**
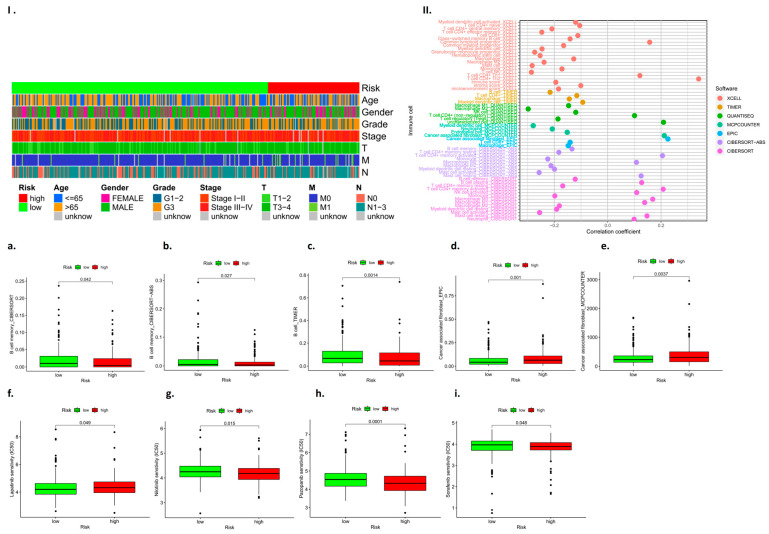
Analysis results for immune cell subcomponent types and tumor drug resistance. (**I**): Heat map of clinically relevant prognostic factors; (**II**): Analysis of prognostic subsets of immune cells; (**a**–**i**): Analysis of immune cell subsets associated with high and low risk prognosis.

We identified RBM10 as a tumor suppressor gene through database screening and PCR assays, and it plays a negative regulatory role in the occurrence and development of lung adenocarcinoma. In this study, RBM10 inactivation was classified as indicating high risk, and high expression of RBM10 was classified as indicating low risk. Based on this feature, we performed a subgroup analysis of clinically relevant factors (such as age, gender, and tumor stage grade) for lung adenocarcinoma. The results indicated that male patients with RBM10 inactivation aged over 65 years with G3 tumor stage were at higher risk (Figure 11I). Some studies [22,23,24,25] demonstrate that this “CMap” resource can be used to find connections among small molecules sharing a mechanism of action, chemical and physiological processes, and diseases and drugs.

The results showed B cell memory, B cell memory-CIBERSORT-ABS, B cell-TIMER, cancer-associated fibroblast-EPIC, etc. (Figure 11a–e). The drug-sensitive cardiac analysis also found that RBM10 had a certain risk correlation with drug resistance to lapatinib, nilotinib, pazopanib, sorafenib, and other drugs (*p* < 0.05) (Figure 11f–i).

### 3.12. In Vitro Experimental Verification Analysis

**Figure 12:**
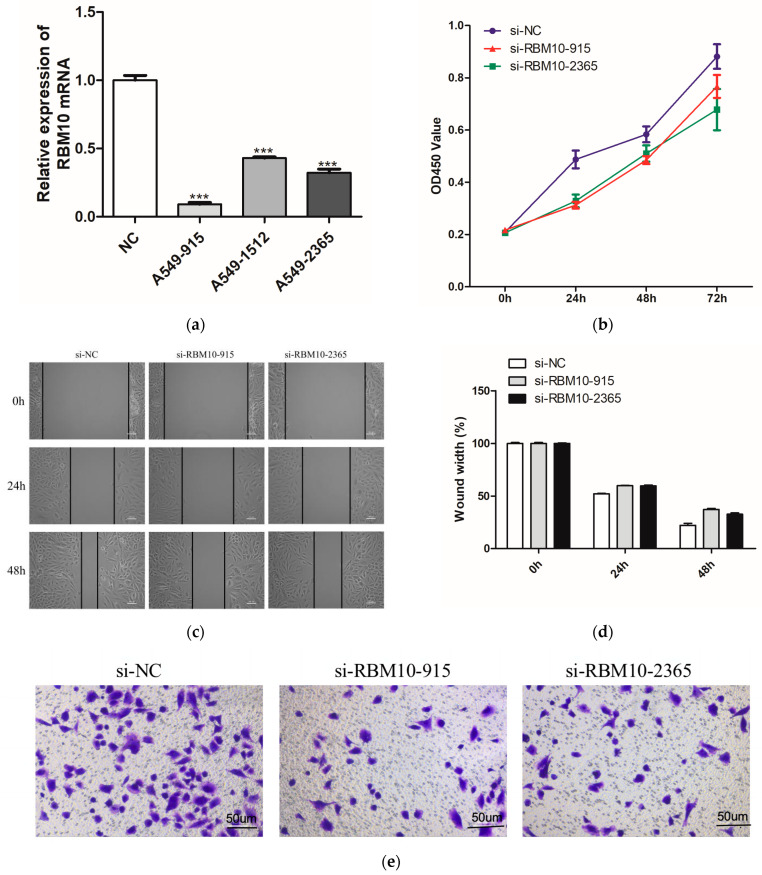

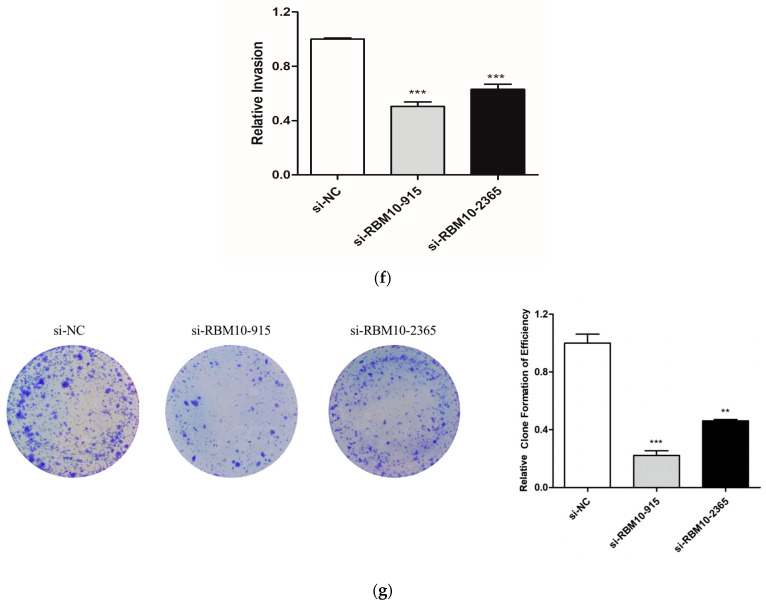
In vitro experimental verification analysis. (**a**): Q-PCR results; (**b**): Cell proliferation was detected by CCK8 for 24h; (**c**): Cell scratch test results; (**d**): Cell migration ability test results; (**e**,**f**): Results of Transwell cell invasion experiment; (**g**): Results of A54 cell cloning experiment (** *p* < 0.05; *** *p* < 0.01).

Further verification in the A549 lung adenocarcinoma cell line showed that the mRNA expression level of RBM10 was lower than that of the control group (Figure 12a). According to CCK8 detection, after 24 h, the proliferation ability of A549 cells in the si-RBM10-915 and si-RBM10-2365 groups significantly decreased compared with that in the si-NC group (Figure 12b). After transfection of si-RBM10-915 and si-RBM10-2365, the migration and invasion ability of the A549 cells significantly decreased (Figure 12c–f). Compared with the control si-NC cells, the cloning rate of the A549 cells after transfection with si-RBM10-915 and si-RBM10-2365 decreased significantly (Figure 12g). DNA methylation is a biological process and an important epigenetic modification. The algorithm was used to predict miRNA target genes, and at the same time, it was combined with the miRNA expression profile, the mRNA expression profile, and the DNA methylation expression profile to find the methylation levels of key miRNAs and their corresponding target genes as well as their corresponding promoter regions [26,27].

## 4. Discussion

Lung adenocarcinoma (LUAD) is the most common histological subtype of non-small-cell lung cancer (NSCLC), accounting for approximately 40% of lung cancers. RBM10 belongs to the RNA-binding motif protein family, which plays an important role in a variety of biological processes [28]. More truncated and missense somatic mutations of RBM10 have been detected in lung adenocarcinoma in recent studies, but the role of RBM10 in lung adenocarcinoma is unclear [29]. Since the occurrence and development of lung adenocarcinoma often involve multiple gene mutations and the activation of and changes in signaling pathways, the specificity of biomarkers is low, and resistance to targeted drug therapy is very relevant for the early diagnosis, treatment, and prognosis of lung cancer; it is important to find new markers for the early diagnosis of lung cancer and analyze their correlation with clinical features [30].

In this study, we collected the expected lung cancer tissues and normal paracancerous tissues of 20 patients who had not received any treatment before surgery, as well as relevant clinical data such as the age, gender, and smoking history of these patients, and further analyzed the risk correlation of independent clinical factors. The results showed that the age of the patients in the high-risk group with RBM10 deletion, their gender, and their tumor stage and grade have certain risk correlations. In the in vitro experiments conducted in this study, we first verified that RBM10 was expressed in the nucleus and cytoplasm of lung adenocarcinoma A549 cells in equal amounts by using an immunofluorescence method, and qRT-PCR showed that the expression of RBM10 in the A549 cell line was significantly lower than that in the control group. Studies [31,32,33,34] have shown that the pathological type of lung cancer of 80% of patients is non-small-cell lung cancer, and lung adenocarcinoma is one of the main tissue types of non-small-cell lung cancer. In the past, platinum-based chemotherapy was used for the treatment of lung adenocarcinoma, but due to the insensitivity of lung adenocarcinoma to chemotherapy and the generation of drug resistance, the therapeutic effect was not sufficient [35]. With the exploration of genomics and molecular biology, researchers have made new discoveries on the formation, invasion, and migration mechanisms of lung adenocarcinoma and re-examined the treatment of non-small-cell lung cancer [36]. In recent years, RNA-binding proteins (RBM) have gradually come into the research field, among which RBM10 and RBM5 can regulate apoptosis-related genes [37]. RBM10 mRNA levels were significantly decreased in lung adenocarcinoma A549 cells. Is RBM10 a potential tumor suppressor gene? As a new potential tumor-related biomarker, whether RBM10 can provide us with new means and strategies for the early diagnosis, targeted drug therapy, and curative effect prediction of lung adenocarcinoma remains to be studied [38,39,40].

The mutation of RBM10 co-exists with the known lung cancer target genes KRAS, EGFR, and PIK3CA, among others [41]. Currently, only patients with an EGFR mutation and ALK gene fusion have clear therapeutic targets clinically, and molecular targeted drugs produced for the corresponding targets, such as imatinib and erlotinib, can be applied in the treatment of these patients [42,43,44]. They can prolong the quality of life of some patients; however, with the progress of treatment, drug resistance to molecularly targeted drugs has also gradually emerged, which has inhibited its clinical therapeutic effect in lung adenocarcinoma patients [45]. As a newly discovered gene related to the progression of lung adenocarcinoma, the specific mechanism of the inactivation and low expression of RBM10 leading to the growth, proliferation, invasion, and metastasis of lung adenocarcinoma cells remains to be further studied and revealed, which can provide new theoretical support for the treatment of lung adenocarcinoma with molecularly targeted drugs. Meanwhile, the research and development of new targeted therapeutic drugs and the discovery of new therapeutic targets for lung adenocarcinoma gene mutations will also be a direction of future research efforts.

## Data Availability

All data can be obtained from authoritative international databases or through the uploaded Supplementary Materials.

## Supplementary Materials

The following supporting information can be downloaded at: https://www.mdpi.com/article/10.3390/biomedicines11071861/s1.
